# 33.3 LEVELS OF AND IMPLICATIONS FOR PERSONAL STIGMA AND MENTAL HEALTH LITERACY IN RELATION TO PSYCHOSIS AMONG YOUNG PEOPLE WITH AND WITHOUT RISK OF DEVELOPING PSYCHOTIC DISORDER

**DOI:** 10.1093/schbul/sby014.139

**Published:** 2018-04-01

**Authors:** Sara Evans-Lacko, Petra Gronholm, Wagner Ribeiro, Kristin Laurens

**Affiliations:** 1 London School of Economics and Political Science; 2 School of Psychology, Australian Catholic University

## Abstract

**Background:**

Much anti-stigma work suggests that reducing stigma and improving mental health literacy could also improve access to care and support for people with psychotic disorders. This is important given that increasing help-seeking, especially during the early stages of psychosis could reduce the substantial delays to care experienced by people with psychotic disorders. Little is known about levels of personal stigma and mental health literacy among young people at-risk of psychotic disorders, whether there are differences between young people with and without elevated risk for psychosis and how this is associated with actual help-seeking for individuals at-risk of developing psychotic disorders.

**Methods:**

We interviewed participants from two existing, ongoing prospective cohorts in the UK and in Brazil. Participants were initially recruited from primary schools. Both samples represent enriched community cohorts (including a greater than average proportion of young people at risk of developing psychotic disorders) in Greater London (n=407) and a similar cohort of young people in Brazil (n=1,500). Participants were presented a vignette depicting a young person with early psychosis symptoms and asked about: recognition of the disorder; intended help-seeking; beliefs about interventions and prevention, stigmatising attitudes and whether they knew someone with a similar problem. We also collected detailed clinical data on psychiatric symptoms (via SDQ [Strengths and Difficulties Questionnaire] in the UK and DAWBA [Development and Well-Being Assessment] in Brazil), presence of psychotic-like experiences, and use of mental health services and personal experiences of seeking support for a mental disorder.

**Results:**

Findings on the relationship between personal stigma and mental health literacy in relation to psychotic disorders, intended help-seeking and actual mental health service use, will be presented among young people with and without risk of developing psychotic disorders in the UK and Brazil.

**Discussion:**

Reducing personal stigma and improving mental health literacy among young people at risk of psychosis who do not yet use clinical services could be important for future help-seeking. Future research should investigate the impact of anti-stigma interventions among young people with and without risk of developing psychotic disorders and how this facilitates help-seeking and support for this vulnerable group.

